# Hypoxia Pathway Proteins As Central Mediators of Metabolism in the Tumor Cells and Their Microenvironment

**DOI:** 10.3389/fimmu.2018.00040

**Published:** 2018-01-29

**Authors:** Sundary Sormendi, Ben Wielockx

**Affiliations:** ^1^Heisenberg Research Group, Institute of Clinical Chemistry and Laboratory Medicine, Technische Universität Dresden, Dresden, Germany

**Keywords:** oxygen sensors, hypoxia, immunity, hypoxia-inducible factor prolyl hydroxylases, glycolysis, lactate

## Abstract

Low oxygen tension or hypoxia is a determining factor in the course of many different processes in animals, including when tissue expansion and cellular metabolism result in high oxygen demands that exceed its supply. This is mainly happening when cells actively proliferate and the proliferating mass becomes distant from the blood vessels, such as in growing tumors. Metabolic alterations in response to hypoxia can be triggered in a direct manner, such as the switch from oxidative phosphorylation to glycolysis or inhibition of fatty acid desaturation. However, as the modulated action of hypoxia-inducible factors or the oxygen sensors (prolyl hydroxylase domain-containing enzymes) can also lead to changes in enzyme expression, these metabolic changes can also be indirect. With this review, we want to summarize our current knowledge of the hypoxia-induced changes in metabolism during cancer development, how they are affected in the tumor cells and in the cells of the microenvironment, most prominently in immune cells.

## Introduction

Metabolism is the set of chemical processes by which energy homeostasis is maintained, allowing cells to adjust to the needs that the surrounding environment demands. By adjusting their metabolic pathway network, cells are able to adapt to nutrients and deprived oxygen availability, as well as to adequately respond to different cell signals. During the past few years, the importance of the metabolic state of a cell and how this exerts differentiation and functionality during physiological and pathological processes has become evident. Indeed, metabolic reprogramming is considered a hall-mark of cancer progression (1, 2). Recently, new progresses in molecular biology and high-throughput molecular analyses revealed that many of the signaling pathways, which are altered by gene mutations can regulate cell metabolism. However, the oncogenic transformation process not only involves cancer cells, but it also alters their tumor microenvironment (TME), which includes stromal and infiltrating immune cells (3). Although in this context their metabolism has received less attention, they signify a rich cell population in many solid tumors. Moreover, the metabolic changes that these cells endure have been shown to have a great impact on their contribution during tumor development. These metabolic changes not only translate in different cell functionality, but they are also important in establishing a pro-tumoral “metabolite crosstalk.” According to this idea, it has been shown that specific excreted metabolites, including lactate (4) are exploited or signal to particular cells. Also the connection between metabolism and signal transduction within the neoplastic area is known. We will, therefore, summarize the most essential and relevant studies in the field of cancer-related metabolism, highlighting the regulating properties of hypoxia pathway proteins.

### Hypoxia Pathway Proteins in Cancer

**Figure 1:**
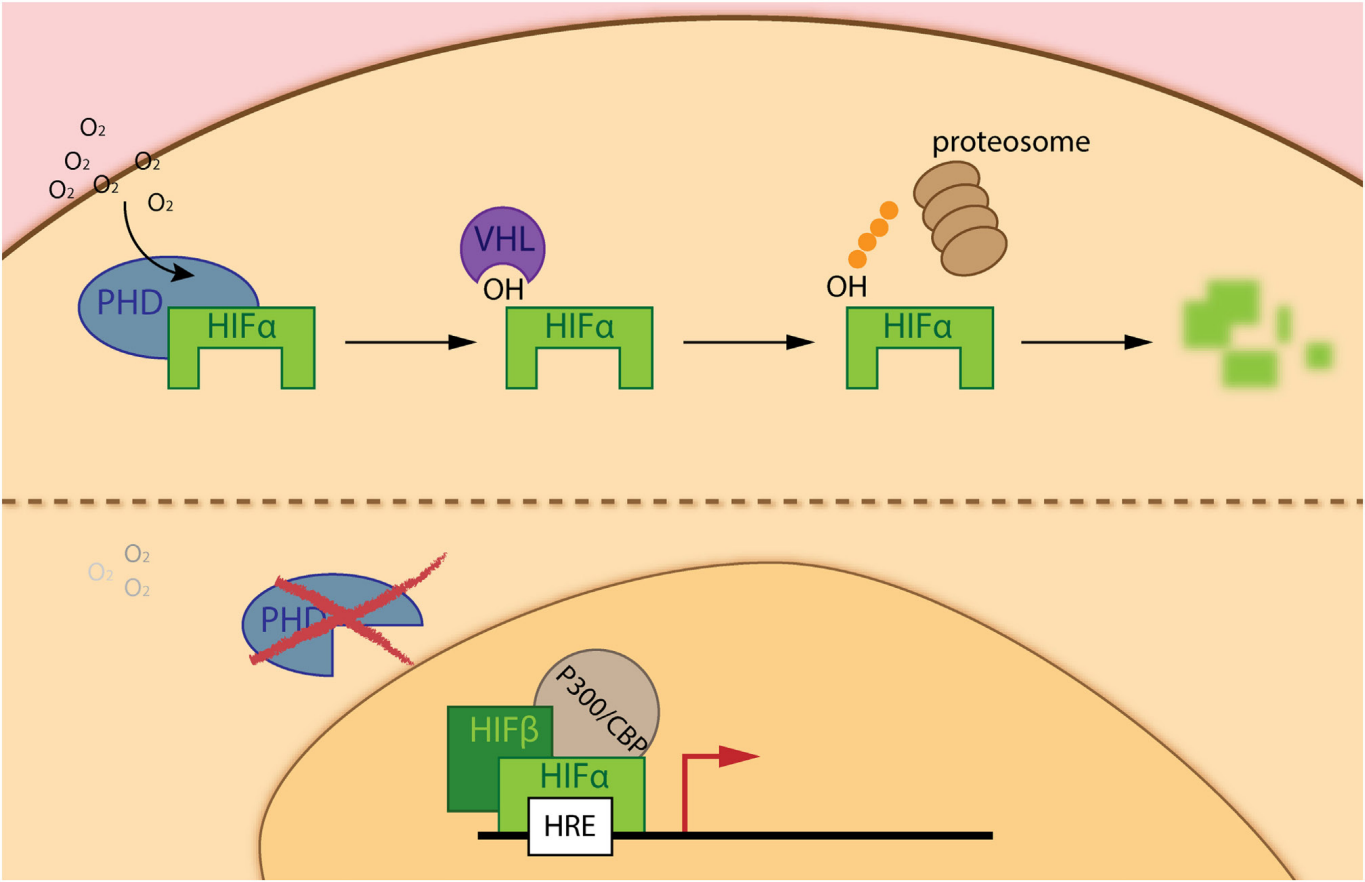
The hypoxia inducible factor (HIF) pathway. Under adequate oxygen pressure, prolyl-hydroxylase domain enzymes (PHDs) hydroxylate two prolyl residues on the α-subunit of the hypoxia-inducible transcription factors (HIF-α). The hydroxylated residues are recognized by the ubiquitin ligase Von Hippel–Lindau, leading to subsequent degradation of HIF-α *via* the proteasome (upper part of the figure). However, during hypoxia (deprived oxygen pressure), PHDs are inactive and HIF-α is able to translocate into the nucleus, interacts with the HIF-β subunit and P300/CBP enabling binding to hypoxia-responsive elements (HRE) in the promotor region of genes implicated in the hypoxia response (lower part of the figure).

Solid tumors are characterized by rapid cell growth that is not equally compensated with a functionally effective and efficient vasculature. This poor vessel irrigation leads to a highly heterogeneous tumor mass with variable oxygen pressure and nutrient levels that cancer cells as well as TME cells need to overcome. Vaupel and colleagues recognized that the partial pressure of oxygen (pO_2_) within human cancers is significantly lower than in surrounding tissue. This so-called intra-tumoral hypoxia is associated with increased risk of local spread, metastasis, and patient mortality (5). Indeed, a complex pathway exists that regulates the adaptive response to hypoxia. The master regulators of the cellular response to hypoxia constitute a heterodimeric complex formed by a constitutively expressed nuclear HIFβ, and a cytoplasmic oxygen-dependent HIFα (HIF-1α, HIF-2α, and HIF-3α) subunit. Stabilization of HIFα is regulated by a group of oxygen and iron dependent enzymes, known as hypoxia-inducible factor (HIF)-prolyl hydroxylase domain enzymes (PHD1–3). Therefore, under physiological oxygen concentrations PHDs hydroxylate two prolyl residues of HIFα, which allows binding of the Von Hippel–Lindau tumor-suppressor protein, leading to subsequent ubiquitination and proteasomal degradation of this alpha subunit. However, in hypoxia PHDs are much less active, allowing gene transcription regulation by the HIF isoforms, with overlapping, distinct or even opposite roles (6) (Figure 1). Since its discovery, regulation of the hypoxia pathway has been strongly related to cancer development. It cannot only modulate survival and proliferation of cancer cells, activation of this pathway can induce angiogenesis, escape from immune-surveillance, epithelial-to-mesenchymal transition, and even distant metastasis (7, 8). Due to the central regulatory role of PHD2 (9) in the hypoxia pathway, several studies have focused on this isoform. In this regard, we were able to demonstrate that loss of PHD2 in tumor cells leads to decreased tumor growth, depending on an anti-proliferative effect of TGFβ activation through matrix metalloproteinases, but not HIF-1α (10, 11). More recently, using a spontaneous breast cancer mouse model, Kuchnio and colleagues showed that PHD2 haplo-deficiency in cancer cells reduce metastasis *via* two mechanisms: (1) by decreasing cancer-associated fibroblasts (CAF) activation due to a reduced secretion of TGFβ by cancer cells, matrix production and contraction by CAFs and (2) by improving vessel normalization (12). As mentioned earlier, newly formed vessels are often disorganized, immature, and leaky. Mice heterozygous for PHD2 are protected from distant metastasis due to endothelial normalization in a HIF2-α-dependent manner (13). Branco-Price and colleagues described that deficiency of HIF-1α in the endothelium diminishes NO synthesis, resulting in retarded tumor cell migration and consequent tumor cell metastasis. However, loss of HIF-2α had a reversed effect (14).

The HIF-pathway proteins not only regulate growth and dissemination of cancer cells, but they also control the tumor-associated immune cells (15, 16). Therefore, many different groups have been focusing on what role the hypoxia pathway proteins play in the two major contrasting forces throughout tumor development: (1) anti-tumor defense and (2) suppression by the immune system. Concerning the latter, tumor-associated macrophages (TAMs) or pro-tumoral macrophages help the tumor to grow. Actually, most of the studies performed relate to the role of the HIF-pathway proteins in TAMs during cancer development. The first studies on this were focused on the role of the HIF transcription factors, revealing that loss of HIF-1α in TAMs increases M2 polarization and pro-angiogenic responses. Moreover, these TAMs overexpress HIF-2α, which correlates with poor patient prognosis (17). In line with this, HIF-2α deficiency in macrophages reduces TAM infiltration into hepatocellular carcinoma in mice (18). In addition, in a transgenic mouse model of breast carcinoma development (MMTV-PyMT), Doedens and colleagues demonstrated that targeted deletion of HIF-1α in macrophages leads to reduced breast tumor growth. Indeed, their work strongly proposes a HIF1-α-dependent macrophage-mediated T cell suppression (19). Furthermore, our research group demonstrated that PHD2 deficiency in myeloid and T cells is a pre-requisite to diminish tumor volume due to increased death of cancer cells (20). Nevertheless, Clever et al. recently reported for the first time a clear role for PHDs in regulating T cell anti-tumoral response. In this study, wild-type and PHD1–3 T cell triple knock-out mice showed similar subcutaneous B16 tumor growth, while the triple PHD KO mice were significantly protected from tumor colonization in the lung (21).

Since hypoxia constitutes one of the hallmarks of solid tumors, and oxygen availability has a direct effect on cell metabolism, it is not surprising that numerous authors have described the reciprocal regulation that HIFs exert on metabolic reprogramming of cancer cells and immune response in the TME and *vice versa* (22–25). In this regard, oxygen not only regulates PHD activity directly (6), CO_2_ production during mitochondrial respiration through the TCA cycle can also suppress HIF activity in high concentrations. The mechanism behind this process still needs to be clarified, but it seems that acidification inhibits protein synthesis (mTOR inhibition) and HIF1 α is extremely sensitive to protein synthesis (26). In addition, ROS production during oxidative metabolism influences HIF activity (27, 28), as well as accumulation of specific immunometabolites such as α-ketoglutarate (α-KG), fumarate, and succinate (29–32).

### Cancer Cell Metabolism

**Figure 2:**
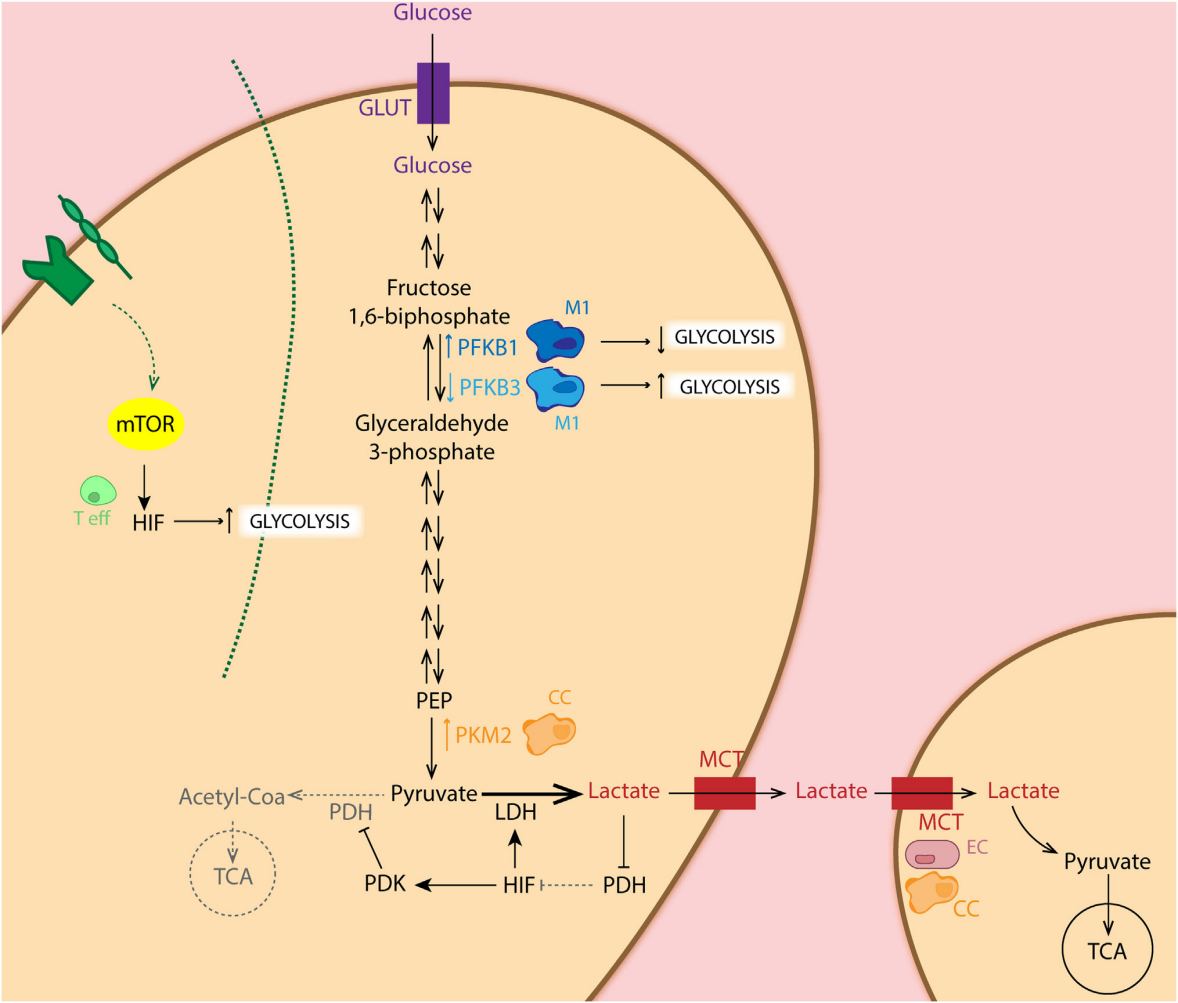
Schematic representation of the glycolytic pathway in different cell types. Metabolic intermediates such as lactate inhibit the PHD function, leading to accumulation of hypoxia-inducible factor (HIF) which regulates expression of several glycolytic enzymes. Importantly, HIF balances glucose metabolism: (1) by inducing expression of LDH, leading to conversion of pyruvate to lactate, (2) as well as dampening the entry of pyruvate in the TCA cycle through inhibition of PDH by pyruvate dehydrogenase kinase (PDK) overexpression. Particularly, cancer cells (CC: orange) show increased expression of PKM2, the limiting rate enzyme of glycolysis, favoring glucose metabolism. CCs as well as endothelial cells (ECs: pink) show increase expression of MCT, facilitating lactate uptake for oxidative phosphorylation. Pro-tumoral M2 macrophages (light blue) present increased expression of phosphofructo-2-kinase/fructose-2,6-biphosphatase 3 (PFKFB3) isoform, enhancing glycolysis in these cells, whereas anti-tumoral M1 (dark blue) express the low activity isoform PFKFB1. Activation of T cells (green) *via* TCR-CD28 leads to enhanced glycolysis essential for their effector functions in an mTOR/HIF-dependent manner.

The Warburg effect is found to be one of the most striking metabolic shifts that healthy normal cells undergo during tumorigenesis (33). This effect of aerobic glycolysis, described by Warburg already in 1920s still forms a hot-topic of tumor metabolism nowadays. This process defines that cancer cells predominantly obtain their energy (in terms of ATP production) through the glycolytic pathway rather than the TCA cycle, even in the presence of adequate oxygen levels (33). But why would cancer cells use glycolysis when energy production is inefficient? Despite the low amount of ATP produced by glycolysis (2 ATP molecules per glucose molecule in glycolysis versus 36 molecules of ATP in TCA), the efficiency of this process relies on faster kinetics of glycolysis, producing a comparable amount of ATP by either form of glucose metabolism during the same period of time (34). This also implies that nutrients are conserved for biosynthesis of nucleic acids, lipids, and amino acids to support cell growth, rather than oxidized in mitochondria for maximal ATP production (35–41). Moreover, this high glycolytic rate entails a great lactate excretion, leading to increased TME acidosis, which alters the tumor stroma interface allowing enhanced invasiveness (42, 43). The presence of variable levels of lactate and hypoxia constitutes one of the main reasons for tumor heterogeneity. Indeed, “metabolic symbiosis” among hypoxic and aerobic cells within the tumor mass has been demonstrated. Lisanti and coworkers described “the Reversed Warburg effect” in which CAFs perform aerobic glycolysis and provide cancer cells with metabolites for oxidative phosphorylation (OxPhos) (44, 45) (Figure 2). In this pro-tumoral “metabolite crosstalk,” lactate produced by hypoxic cells is taken up by aerobic cells, which use it as their principal substrate for OxPhos. Lactate recycling is not new, and is well known from the Cori cycle in the liver (46). Sonveaux et al. showed that human cancer cells cultured under hypoxic conditions convert glucose to lactate and excrete it, while aerobic cancer cells take this lactate back up *via* monocarboxylate transporter 1 (MCT1) and utilize it for OxPhos (4). Another important glycolysis-related enzyme is pyruvate kinase (PK), which catalyzes the final glycolytic reaction. Therefore, reduction of PK activity causes a build-up of glycolytic intermediates that are redirected toward biosynthesis. Elevated expression of the isoform PKM2 has been demonstrated in several types of cancer, including colon, kidney, lung, and breast (47). Several studies have shown that PKM2 directly regulates the Warburg effect, since the knock-out of this enzyme reduces glucose uptake and lactate production, increasing oxygen consumption, and finally reducing tumorigenesis (48–50). In addition to this, Luo et al. reported that hydroxylation of PKM2 by PHD3 allows its binding to HIF-1α, enhancing expression of HIF-1α targeted genes (51). In addition, HIF-1α restricts OXPHOS and regulates the expression of pyruvate dehydrogenase kinase (PDK), an enzyme that phosphorylates and inactivates pyruvate dehydrogenase. The latter limits pyruvate utilization for OxPhos (52). Furthermore, active Akt2 accumulates in the mitochondria during hypoxia and phosphorylates pyruvate dehydrogenase kinase 1 (PDK1) on Thr346 to inactivate the pyruvate dehydrogenase complex (53). Regarding the HIF pathway, cancer cells present frequent activation of the PI3K–mTOR axis, which functions as a nutrient sensor pathway. mTOR activation favors HIFα activity and promotes tumor angiogenesis. Thus, it has been shown that loss of the mTOR inhibitor TSC2 (tuberous sclerosis complex 2 protein) results in the accumulation of HIF-1α and increased expression VEGF (54). Another study relates mTOR-mediated regulation of HIF-1α to the pathogenesis and increased angiogenesis in chronic myelogenous leukemia (55) (Figure 2).

Previous studies have shown that specific metabolites are able to directly regulate the hypoxia pathway. Therefore, loss-of-function mutations of the tumor suppressor genes encoding the succinate dehydrogenase complex and fumarate hydratase lead to the accumulation of succinate or fumarate, resulting in HIF stabilization through inhibition of PHDs (56). Also other intracellular metabolites, such as pyruvate, lactate and oxaloacetate block PHD-mediated inhibition of HIF-1α underlying its prominent basal activity, commonly seen in many highly glycolytic cancer cells. This suggests that enhancement of HIF-1 by glucose metabolites may constitute a feed-forward signaling mechanism involved in malignant progression (57). In addition, isocitrate dehydrogenases 1 and 2 (IDH1 and IDH2) are frequently mutated in cancer. These enzymes function at the intersection of different processes, including oxygen-sensing signal transduction, cellular defense against oxidative stress, oxidative respiration, and cellular metabolism in lipid synthesis. The mutated forms of IDHs (58) produce 2-HG instead of α-KG essential for PHD function (59). Indeed, it has been shown that 2-HG can either inhibit or activate PHD-driven hydroxylation of HIF in an enantiomer-specific way (60).

Since the discovery of the Warburg effect, mitochondrial dysfunction was designated as a metabolic hallmark of cancer cells. However, earlier studies provided genetic evidence that mitochondrial metabolism is essential for tumorigenesis (61–63). Indeed, cancer cells generate an abundant amount of NADPH in the mitochondria and the cytosol to sustain high antioxidant activity and prevent the build-up of potentially detrimental ROS (64, 65).

**Figure 3:**
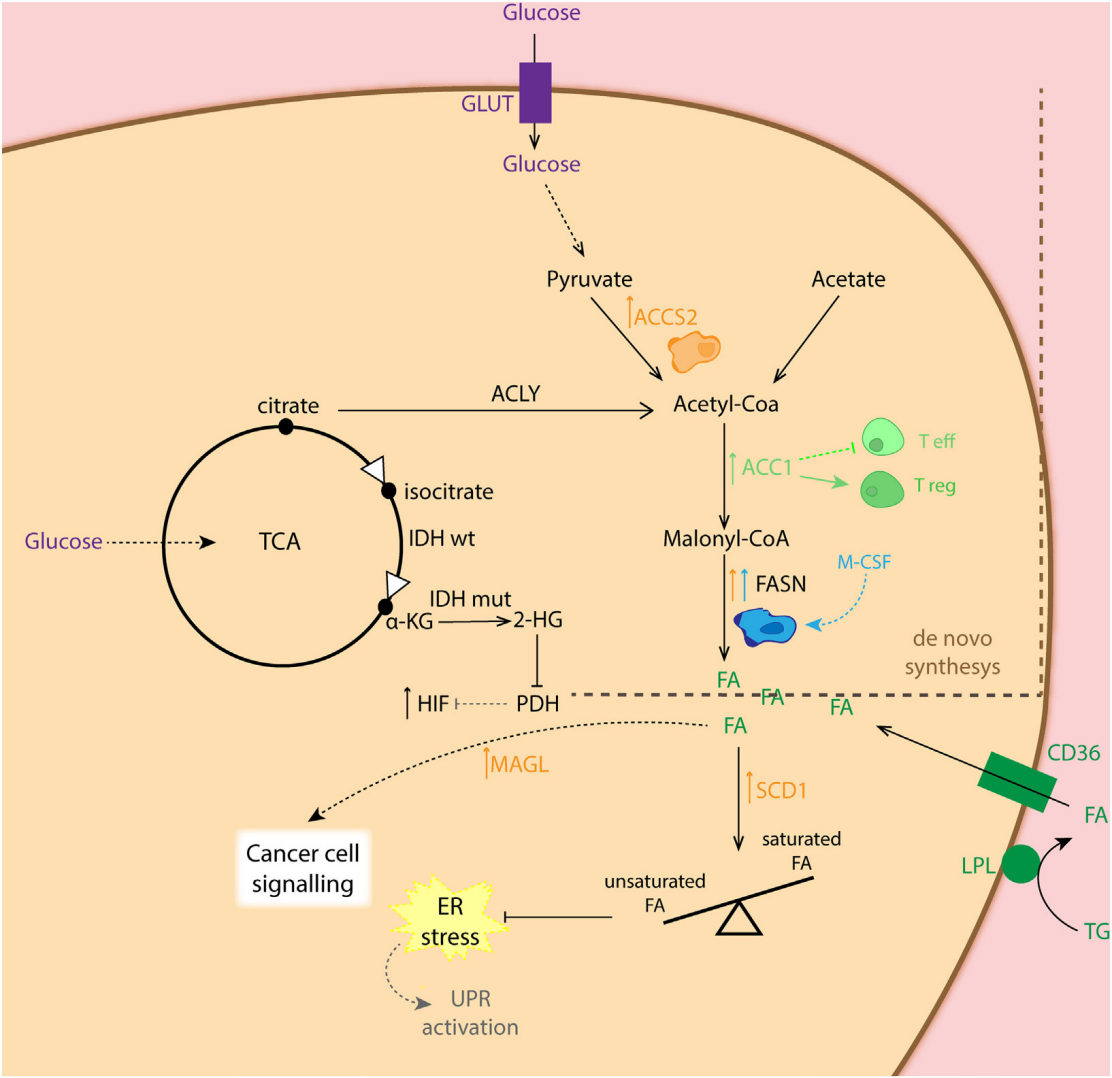
Schematic representation of the fatty acid (FA) synthesis in different cell types. Cancer cells (CC: orange) show increased expression of ACCS2 favoring conversion of pyruvate to acetyl-coA as well as fatty acid synthase (FASN) for conversion of malonyl-coA to FA. The latter, is also overexpressed in M2 macrophages (blue). CCs can also obtain FA from exogenous sources *via* converting triglycerides (TG) into FA, which are taken up by the CD36 transporter. Overexpression of monoacylglycerol lipase (MAGL) in CC is related to synthesis of lipids involved in cell signaling. Increased expression of SCD1 in CC balances higher presence of unsaturated FA essential for maintenance of ER homeostasis. Enhanced expression of ACC1 shifts T cell differentiation toward T regulatory cells (Treg: dark green).

Although anaerobic glycolysis is an acclaimed feature of cancer cells, this is not the only metabolic alteration in the transformed cells. In fact, for tumor cells to proliferate, fatty acid (FA) synthesis (for membrane biogenesis) as well as glutaminolysis (for amino acid precursors) has been reported to be affected during tumorigenesis (37, 66, 67). It has been shown that lipid production is critical for cancer cell survival, while the expression of the central lipogenic enzyme fatty acid synthase (FASN) is strongly correlated with cancer progression (68, 69). FAs used for cancer cells during lipogenesis can be endogenously derived from citrate in the TCA cycle, but they can also be seized from exogenous sources. To obtain free FA from circulation, lipoprotein lipase (LPL) hydrolyzes circulating triglycerides. Then, free FAs are imported into the cell *via* the FA translocase CD36. Both proteins LPL and CD36 are widely expressed in breast, liposarcoma, and prostate tumor samples (70) (Figure 3). In addition to this, lipid metabolism plays an important role in preventing ER stress, as it appears that balancing saturated and unsaturated lipid species are required, due to the lipotoxic effects of the former. Therefore, desaturation of *de novo* synthesized lipids by oxygen-dependent stearyl-Coenzyme A (CoA) desaturases (SCDs) plays a critical role for cancer cell survival. In this regard, SCD1-mediated lipid desaturation has been found to be a critical determinant of cancer cell survival downstream SREBP transcription factors (which are regulated by the mTORC1 pathway) (71). In fact, unsaturated lipid deficiency of hypoxic cells has been shown to cause cell death by ER stress and activation of the unfolded protein response in an mTORC1-dependent manner (72, 73). Another important lipid-metabolism-related enzyme is acetyl-CoA synthase 2 (ACSS2). ACSS2 converts acetate to acetyl-CoA, which is used as a nutritional source by cancer cells supporting biosynthesis of membrane phospholipids. Moreover, it is an epigenetic regulator in histone acetylation. It was also shown that hypoxia enhances the expression of ACSS2, which has been related to poor prognosis in breast cancer patients (74). The importance of lipid metabolism alterations in cancer cells relays not only on the role of lipids for biogenesis but also on their capacity to signal. In this respect, it has been shown that in human cancer cells as well as primary tumors, monoacylglycerol lipase (MAGL) is vastly overexpressed. This enzyme regulates a FA network that drives oncogenic signaling lipids, which promotes migration, invasion, survival, and *in vivo* tumor growth (75).

**Figure 4:**
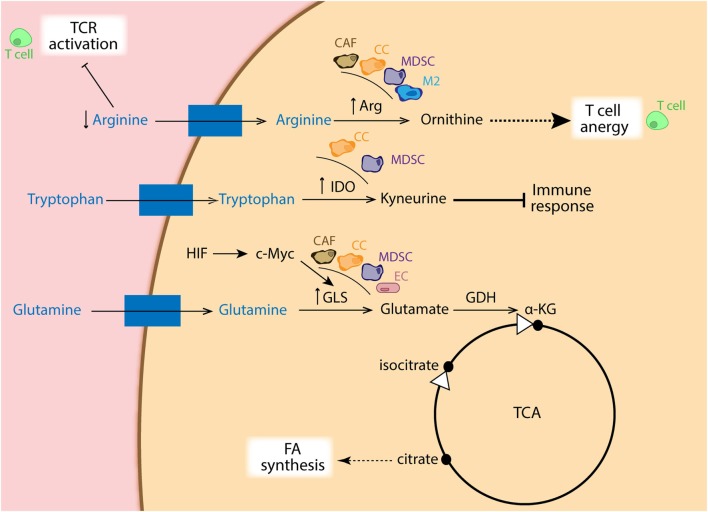
Schematic representation of amino acid metabolism in different cell types. Cancer cells (CCs: orange), cancer-associated fibroblasts (CAFs: brown), myeloid-derived suppressor cells (MDSCs: purple), as well as M2 macrophages (blue) show increased expression of arginase, depleting the surrounding environment of arginine, which is essential for TCR activation in T cells (green). Ornithine coming from arginase metabolism also dampens T cell activation. Moreover, increased expression of indoleamine-2,3-dioxygenase (IDO) in CC and MDSCs favors catabolism of tryptophan into kyneurine, with immune suppressive effects. In addition, overexpression of GLS *via* HIF/c-Myc regulation in CA, CAFs, MDSCs, and endothelial cells (ECs: pink) generates glutamate, which is used to replenish intermediates of the TCA cycle, favoring FA synthesis from citrate.

As mention before, tumors are glutamine addicted. Cancer cells display high rates of glutaminolysis in order to obtain several precursors needed for supporting robust proliferation. In this regard, c-Myc has been shown to directly upregulate glutamine-metabolizing enzymes, such as glutaminase, which leads to fast integration of nitrogens and carbons in the anabolic network (76–78). Interestingly, it has been reported that the c-Myc function is directly regulated by both HIF-1α and HIF-2α (79, 80). The reductive metabolism of glutamine, mediated by IDH1, adds extensively to lipogenesis in cancer cells (81) (Figure 4), and which is partially facilitated by an increase in PDK1 (53, 82) and c-Myc (76, 83) in a HIF1-dependent manner, but it is primarily determined by the relative abundance of citrate and α-KG (83, 84). In addition, Kynurenine is another oncometabolite, which was defined as a tryptophan metabolite made from indoleamine-2,3-dioxygenase (IDO) (85), and known for its robust immunosuppressive effects (86) (Figure 4).

In addition to this, metabolic fitness relates to an increased HIF signaling in tumor cells, which permits the cancer cells to strive better for crucial metabolites, such as glucose and glutamine, than the stromal cells (87). This competition for nutrients has been demonstrated in the exhaustion of tumor-associated lymphocytes, suggesting a metabolic associated immune suppression (88, 89).

### Tumor Microenvironment

**Figure 5:**
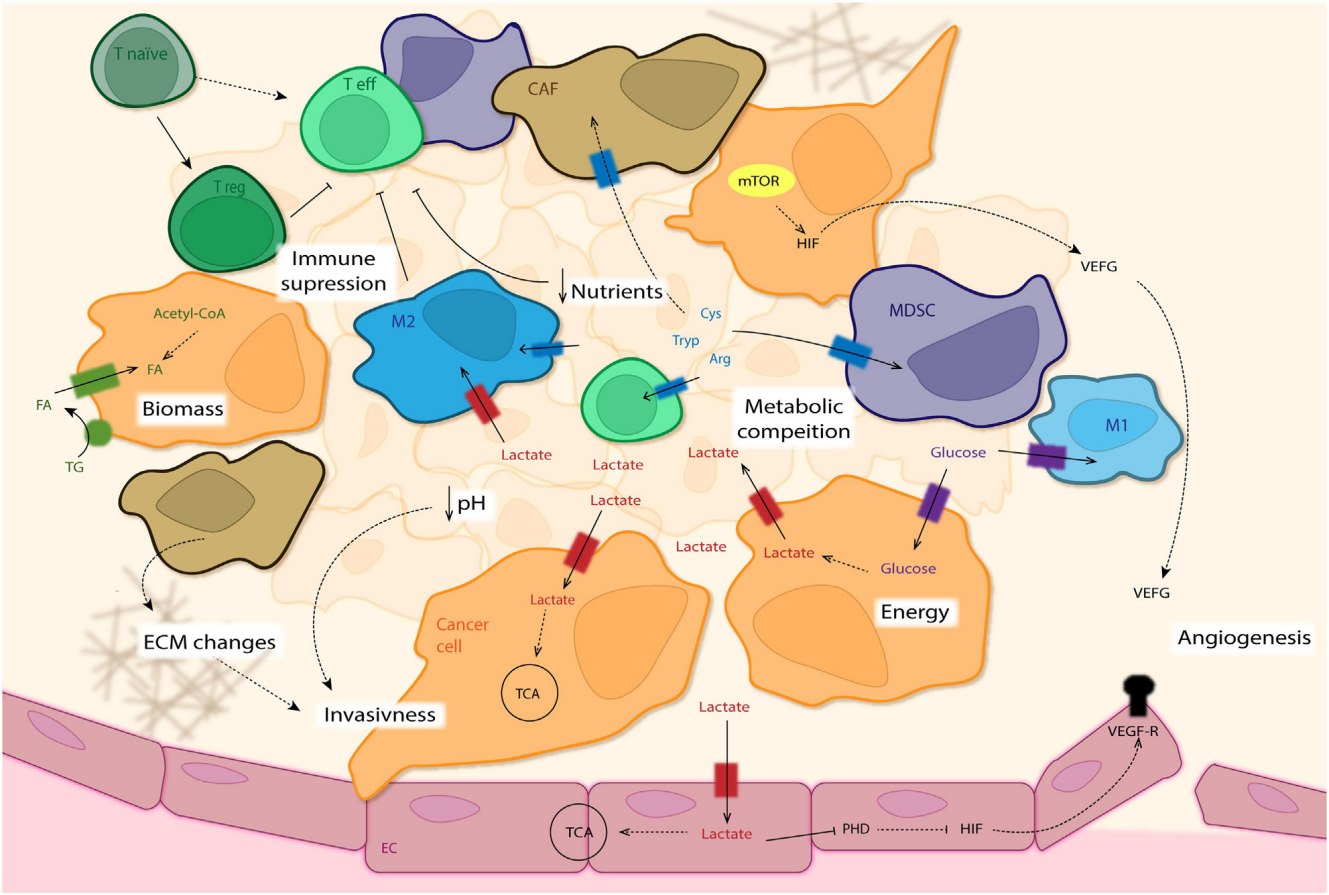
The tumor microenvironment (TME). This overview summarizes the intricate interactions of the different cells types that constitute the tumor mass. Some cancer cells (CC: orange) use glycolysis as main source for energy production (independently of oxygen availability—Warburg effect), excreting lactate and leading to acidification of the surrounding area, which finally promotes invasiveness of CC. Other CCs as well as M2 macrophages (blue) and endothelial cells (ECs: pink) with access to adequate levels of oxygen use this lactate for oxidative phosphorylation (OxPhos). Lactate in ECs leads to inhibition of PHD and subsequent activation of HIF, enhancing expression of VEGF-Rs (among others) and promoting angiogenesis. Uptake of fatty acids (FA) as well as *de novo* synthesis is used for new biomass required for fast CC proliferation. Next to amino acid depletion in the tumor mass by M2 cells, cancer-associated fibroblasts (CAF: brown) and myeloid-derived suppressor cells (MDSCs: purple) establish a metabolic competition between pro- and anti-tumor forces present in the TME. The absence of nutrients needed for T effector cell activation (Teff: light green) leads to their anergy, with a differentiation shift toward T regulatory cells (Treg: dark green). The lack of nutrients leads to activation of the mTOR pathway in CCs and production of VEGF (among others) *via* HIF regulation, promoting angiogenesis.

Although tumor cells have been the main subject of study in cancer research, stromal cells and infiltrating immune cells have gained great interest, over the past years. Furthermore, the intriguing crosstalk of tumor cells with the TME or even different components of the TME among each other (as introduced in the previous section), regulate a vast amount of processes during tumor development (3). In this section, we will discuss several metabolic adaptations of different cell types of the TME (Figure 5).

#### Stromal Cells

##### Endothelial Cells (ECs)

Endothelial cells are the best characterized cells of the TME. Despite the fact that ECs dispose of immediate access to oxygen in the blood, ECs are highly glycolytic, generating up to 85% of their ATP *via* glycolysis. Indeed, their glycolytic rate is comparable to that of cancer cells, and increases even during proliferation (90). Rather than acting as a bioenergetic power source, it has been reported that through the production of pro-angiogenic reactive oxygen species (ROS) mitochondria in ECs have a signaling function (91). In this regard, also the important role of the pentose phosphate pathway (PPP) as a weight against oxidative stress is noteworthy, as it controls redox homeostasis through NADPH production, together with ribose-5-phosphate for synthesis of lipids nucleotides and amino acids (92). Although glutamine and FA metabolism for ATP production in ECs is still under debate, glutaminolysis has been described to be essential for EC proliferation, as inhibition of glutaminase induces their senescence (93, 94). Also cholesterol synthesis has been shown to be crucial for vessel sprouting, as it enables the development of lipid rafts required for proper membrane localization and signaling of the VEGF receptors (95). At this point, it is important to note that VEGF creates a tip cell signal, required for vessel sprouting, a process during which ECs display exclusive patterns of cellular metabolism with high rates of glycolysis and reliant on FAO for nucleic acid synthesis and proliferation (94, 96). Since tip cells are located far from functional vessels, their activity is also regulated *via* the hypoxia pathway (97). In this regard, it has been shown that deletion of endothelial HIF-2α enhances angiogenesis, although the vessels were more disorganized and hypoxic (98, 99). In addition, PHD2+/− mice showed increased HIF-α stabilization, and normalization of the endothelium, increased oxygenation and reduced secondary metastasis (13). On top of this, it has been reported that the HIF subunits have opposing roles when it comes to the permeability of the endothelium. HIF-1α deficiency in ECs hampers tumor cell migration through endothelial layers, while loss of HIF-2α enhances tumor cell migration and metastasis (14). These contrasting effects may be directly related to the dramatic differences in inducible nitric oxide synthase (iNOS) expression in both deficient lines. In addition to this, a pro-tumoral “metabolite crosstalk” between EC and cancer cells has been reported. In this regard, Boidot et al. identified a direct link between the function of p53 and MCT1 expression, regulating the influx of lactate produced by cancer cells into EC (100). Later studies from the same group showed that internalized lactate through MCT1 by EC promote tumor angiogenesis through PHD2 inhibition, and activating HIF1 (101, 102). The same mechanism has been reported to trigger IκBα degradation, stimulating an autocrine pro-angiogenic NFκB/IL-8 pathway, and finally driving cell migration and tube formation (103). Furthermore, the receptor tyrosine kinases AXL, TIE2, and VEGFR-2 is activated by lactate in order to promote angiogenesis (104).

##### Fibroblasts

Another important stromal cell of the TME is the CAF. The importance of CAFs relies not only on their ability to produce growth factors and chemokines regulating other stromal cells and cancer cells but also in their capacity to modify the extracellular matrix (ECM), facilitating tumor angiogenesis and invasiveness (105). CAFs is actually a mix of myofibroblast-like cells that ascend different types of cells, including fibroblasts, bone-marrow-derived stromal cells, ECs, and adipocytes (106–108). Despite their relevance in regulating tumor development, studies on the metabolism of CAFs have been limited. Interestingly, proliferating fibroblasts produce biomass for a next proliferation, while quiescent fibroblasts use biomass to replace oxidized lipids and degraded proteins, as well as synthesis of ECM proteins. Hypoxia pathway proteins are an important stimulus of this process, since they increase the expression of remodeling enzymes leading to increased tumor rigorousness and enhanced metastasis (109). In this respect, reduced PHD2 activity led to a diminished CAF-induced ECM remodeling and diminished metastasis (12, 110). Independent of their activation state, healthy fibroblasts incorporate glucose carbons in the TCA cycle at comparable rates (111). Fibroblasts have also been described to replenish intermediates of the TCA by a process known as anaplerosis. In particular, anaplerotic flux from pyruvate to oxaloacetate *via* pyruvate carboxylase in quiescent fibroblasts ensures continuity of the TCA cycle, whereas proliferating fibroblasts primarily use glutamine for anaplerosis. Like cancer cells, proliferating fibroblasts rely on PPP for biosynthesis. By contrast, quiescent fibroblasts generate NADPH *via* the PPP, which is essential for their survival (111). Moreover, CAFs also perform FA synthesis, essential for their proliferation (112). Production of ROS by cancer cells inhibits PHD2 (with subsequent gain of function of HIF-1α) and enhances NO production by CAFs. This finally leads to dysfunctional mitochondria that are mitophaged, forcing CAFs to rely on glycolysis for ATP production, with accompanied increase in lactate production (113). High lactate production together with amino acids and keton bodies supply cancer cells with high-energy nutrients for oxidative metabolism in tumor oxygenated areas (114, 115). In addition, loss of HIF-1α in fibroblasts leads to vascular normalization, decreases hypoxia but increases breast cancer development (116). HIF1α-driven aerobic glycolysis in stromal cells supports cancer cell growth *via* the paracrine production of nutrients (such as lactate), which cancer cells can recycle (4, 102, 117, 118). As we will discuss in the following section, arginases (ArgI and ArgII) are important for immune suppression in the tumor, converting l-arginine to ornithine, resulting in T cell anergy and reduced anti-tumor response (119). CAFs that are localized to hypoxic regions in pancreatic tumors express high levels of ArgII, suggesting for CAF-mediated immunosuppression (120).

#### Immune Cells

Although numerous studies have indicated the involvement of almost every immune cells type, macrophages have been by far the most studied immune cell type during cancer development. As mentioned before, the importance of metabolism in regulating immune cell phenotype and function and its impact during tumor development is well known (121). Indeed, during the past years, a new field of study has emerged, focusing on the metabolism of the immune system also known as immunometabolism. This new area of research studies how changes in cell metabolism regulate the immune system during homeostasis as well as during inflammatory processes, including tumor-associated inflammation. Indeed, how metabolic changes in immune cells during tumor development regulate the contribution of these cells to disease progression has been the center of a great number of studies. As mentioned before, high levels of glycolysis but hampered angiogenesis inside hypoxic tumor areas can result in near glucose depletion and accumulation of waste products such as lactate. Hence, anti-tumoral immune cells infiltrating TME face significant metabolic challenges to mount and sustain against the tumor. In this regard, T cells have been extensively studied as the main force fighting tumor growth, characterized by particular metabolic shifts according to their activation state (Figure 5).

##### T Cells

Resting naïve T cells require low amounts of glucose, amino acids, and FAs to sustain basic energetic and minimal replacement demands. More than 90% of their ATP production comes from FAO and OxPhos, whereas glutaminolysis and PPP contribute to biosynthesis purposes. Upon activation, T cells increase glucose and glutamine catabolism for nucleotide and lipid synthesis that are essential for cell growth, while OxPhos for ATP production is maintained (122–125) (Figure 5). Indeed, glycolysis has been described to be essential for T cell effector functions, since its impairment suppresses proliferation. TCR–CD28 co-stimulation triggers the shift from naïve to effector T (Teff) cells through PI3K/Akt/mTOR pathway, and activation of cMyc and HIF-1α transcription factors. This promotes glycolytic gene expression and post-translational modification essential to drive aerobic glycolysis and amino acid metabolism in Teff cells, while suppressing catabolic FAO for ATP (122, 126–128). On the other hand, T regulatory (Treg) cells orchestrate a pro-tumoral environment by inhibiting effector T cell responses in the tumor area. Contrary to Teff, Treg cells rely on both FAO and OxPhos for ATP upon activation (127, 129, 130). This metabolic state allows Treg cells to survive tumor conditions and exert their immunosuppressive effect, whereas anti-tumor effector T cells would face impair TCR signaling due to lack of glucose (122, 123, 129–131). Indeed, Treg expansion has been linked to activation of the nutrient stress sensor AMPK. Thus, when the AMP:ATP ratio increases due to the lack of nutrients, AMPK favors oxidative catabolic pathways (132). This metabolic shift implies that AMPK can immediately impact the balance of Teff and Treg cells *via* mTORC1 inhibition (133). Upon CD3/CD28 activation, T cells accumulate metabolites involved in anabolic pathways increasing FAS (122). In addition, it has been shown that mTORC impairment compromise *de novo* lipid synthesis in T cells through induction of the transcription factor SREBP (134). The importance of lipid metabolism in T cell biology has been also reported at the level of the FAS limiting rate enzyme ACC1, which deletion interferes with differentiation of naïve to effector T cells (135). However, ACC1 deletion did not affect the ability of naïve T cells to proliferate and differentiate into Treg (135, 136), suggesting FAS as an important metabolic checkpoint during activation-induced differentiation into Teff cells. Similar to cancer cells, PI3K–mTOR axis stimulates HIFα activity, downstream of the TCR activation (137, 138). Also, IL6 stimulation of T cells leads to JAK–STAT pathway increased transcription of HIF mRNA (126, 139). As mentioned before, metabolites themselves can also act as signaling molecules. In this regard, decreased flux through the TCA cycle may lower succinate levels. It has indeed been shown that succinate can stabilize HIF-1α, inducing transcription of several inflammatory cytokines (32). Although there are not many studies on the role of the HIF-pathway in T cells during tumorigenesis, previous studies have reported a role for this pathway during T cell-mediated inflammation and differentiation (Th17 and Treg balance). Dang et al. reported that HIF-1α enhances Th17 differentiation by direct transcriptional activation of RORγt, while inducing FoxP3 proteasomal degradation and dampening Treg differentiation (126). During Th17 cell development, glycolysis rate is increased through mTOR–HIF-1α signaling induction (127). The tumor protecting or promoting role of Th17 is still controversial due to its differently described phenotypes [for review, see Ref. (140)].

Moreover, it has been reported that T cell activation is blocked due to disruption of the electron transport chain leading to impaired mitochondrial ROS production (141). Apart from this, the hypoxic environment within the tumor area may protect tumor cells from anti-tumor immunity by HIF-1α-dependent upregulation of PD-L1 on cancer cells, which inhibits PD-1 expressing T effector cells (142). Moreover, high lactate levels in the tumor area have been shown to suppress the PI3K/Akt/mTOR pathway inhibiting glycolysis, finally leading to impaired T cells (128, 143, 144). Glycolysis inhibition can lead to increase expression of PD-1, which is associated with T cell exhaustion and non-responsiveness through inhibition of TCR and CD28-mediated co-stimulation, helping the tumor to escape immune surveillance (145). Also tryptophan has gotten attention as a limiting amino acid in T cell activation. Tryptophan metabolism is mainly regulated by IDO, highly expressed by cancers, and in fact correlated with poor prognosis (146) (Figure 4). Using a mouse sarcoma model, Chang et al. showed that glucose restricts T cells, leading to hampered mTOR activity, glycolytic capacity, and INFγ production. The result is enhanced tumor progression. Checkpoint blockade using antibodies against CTLA4 PD1 and PDL1 restore all previous changes (88). Recent work supports the hypothesis that lactic acid blunts the immune response mediated by T and NK cells (147).

##### Macrophages

Historically, macrophages have been classified as M1 (classically activated) and M2 (alternatively activated) according to their pro- or anti-inflammatory state, respectively. However, more recently, the idea of a multidimensional spectrum rather than dual macrophage activated states has emerged (148, 149). Since specific stimuli induce specific functional outcomes, it is expected that different states of polarization present a particular metabolism. M1-phenotyped macrophages are highly glycolytic and characterized by an increased induction of the strong glycolytic enzyme 6-phosphofructo-2-kinase/fructose-2,6-biphosphatase 3 isoform (PFKFB3) (150), conferring them an energetic advantage in hypoxic regions (151). This glycolytic state is mediated by the Akt/mTOR/HIF-1α pathway (32) and has been shown to induce TNF expression (152). The latter suggests a direct regulation of the inflammatory phenotype of macrophages depending on the glycolytic pathway. In relation to their anti-inflammatory role, M1 macrophages also use PPP and malic enzyme in order to produce high amounts of NO and ROS for killing pathogens, as well as NADPH to protect themselves from this high oxidative burst (153). On the other hand, M2 macrophages present high rates of FAO and OxPhos, with low glycolytic activity due to the expression of the weak glycolytic activator PFKFB1 isoform (154, 155) (Figure 2). O’Neill et al. showed that M2 polarization upon IL-4 signaling stimulates mitobiogenesis by upregulating PGCβ, enhancing the metabolic switch to FAO (155). Also, M2 macrophages reduce PPP flux and GSH *via* induction of carbohydrate kinase-like protein (CARKL) (156). Traditionally, TAMs have been related to a more M2 anti-inflammatory and pro-tumor phenotype. However, the fluctuating levels of lactate and oxygen in the heterogeneous tumor mass induce differential macrophage responses depending on their functional plasticity in tumor. In general, a lactic acid-induced polarization to M2 has been reported, inducing an immunosuppressive and tissue remodeling phenotype. This is characterized by the production of VEGF and arginase in a HIF-1α dependent manner (43, 121). Also, TAMs respond and adapt to different oxygen levels through activation of the HIF pathway. In this regard, it has been shown that expression of HIF-1α has a protective role in hypoxic areas, since loss of this transcription factor leads to decrease expression of IL-6, TNF, and iNOS, as well as increased CD206, all of them characteristic markers of the M2 phenotype (15, 17). TAMs are also characterized by high expression of arginase enzyme (induced by the high lactate levels in the TME). This has been shown to impair anti-tumor T cell function due to depletion of the arginine pool required for NO and protein synthesis leading to TCR function impairment (19, 43, 157, 158). Apart from this, recent studies have described the importance of iron metabolism in macrophage polarization. M1 macrophages express reduced levels of ferroportin, the iron transporter, but high levels of H-ferritin involved in iron storage, whereas M2 macrophages present the opposite profile. Therefore, iron sequestration in M1 macrophages is believed to restrict both bacterial and tumor growth, while M2 macrophages release iron, which promotes tissue repair and tumor cell proliferation (159, 160). In addition, iron constitutes a cofactor of the PHD enzymes in the hypoxia pathway. Therefore, intracellular iron levels directly regulate HIF-1α stability crucial for the survival and pro-tumor function of TAMs (161). In a tumor setting, TAMs also undergo changes in their lipid profile. It has been shown that M-CSF secreted from tumor cells leads to enhanced expression of FASN in macrophages, which polarize to an IL-10 expressing pro-tumoral phenotype (162).

##### Myeloid-Derived Suppressor Cells (MDSCs)

Another important immune cell type that has recently gained great attention is the MDSCs that, as its name indicates, is functionally defined by its potent immunosuppressive activity in both innate and adaptive immunity. This cell population comprises two major subsets: monocytic MDSCs (M-MDSCs) and polymorphonuclear MDSCs (PMN-MDSCs) (163). G-CSF has been described to play a critical role in differentiating and mobilizing bone marrow granulocytic precursors within tumors (164); whereas depending on the magnitude and context of the stimulus, GM-CSF can induce accumulation of these suppressor subsets thereby inhibiting proliferation as well as anti-tumor ability of neu-specific T cells (165, 166). In addition, it has been shown that IL-4Rα activation through IL-4 and IL-13 exposure evokes MDSCs suppressive mechanism in a STAT6-dependent manner (167, 168). MDSCs promote immune dysfunction by using different mechanisms, either directly *via* impairment of T cell amino acid metabolism or through regulation of oxidative stress, which finally interferes with T cell viability, migration, and activation. Also, MDSC are able to indirectly induce other immune regulatory cells, such as Treg cells and TAMs (169–171). The same as macrophages, MDSCs show high expression of arginase, depleting arginine from the TME essential for TCR activation and T cell proliferation (172, 173). MDSCs also sequester the amino acid cysteine, which is indispensable for T-cell activation (174) and expresses IDO enzyme for tryptophan catabolism (175, 176). Deprivation of the later has been shown to induce expansion of the Treg cell population (177). Combined expression of nitric oxide synthase, arginase, and NADPH oxidase confers MDSC important regulators of oxidative stress in TME (178–181). Therefore, presence of RNI (mainly derived from M-MDSC), and ROI (mainly from PMN-MDSC) downregulates TCR and IL2 receptor signaling, inhibiting T cell activation and proliferation (170). In addition to this, MDSCs show enhanced FA uptake and high expression of FAO enzymes, accompanied by an increased mitochondrial mass and oxygen consumption rate (182). Corzo et al. described the role of the hypoxia pathway in MDSCs, with HIF-1α as main responsible for MDSC differentiation and function in TME (183). In addition, HIF-1α-mediated expression of PD-L1 is essential for mediating MDSC immune suppression (as discussed in the previous sections) (184). Also hypoxia can enhance MDSC migration to the tumor site *via* HIF-1α-mediated production of chemokines (185, 186). Hypoxia also influences seeding of MDSCs in the pre-metastatic niche by stimulating increased secretion of lysyl oxidase (187–189). This process drives ECM remodeling in the metastatic niche and suppresses NK anti-tumor response (188).

##### Neutrophils

During the past few years, the presence of tumor-associated neutrophils (TANs) has gained attention due to their pivotal role in tumor development. Indeed, a dual effect has been proposed for TANs during onset (190). In this regard, Fridlender et al. showed that in the absence of TGFβ, TANs encourage Teff response and anti-tumor activity, whereas in the presence of TGFβ they exhibit tumor promoting activity (191). Neutrophils comprise a significant proportion of the inflammatory infiltrate in cancerous lesions and high levels of blood neutrophils were observed in patients suffering from advanced stage tumors (192). In many cancer types, such as bronchoalveolar carcinoma (193), metastatic melanoma (192), and andrenal carcinoma (194), neutrophil accumulation was associated with increased aggressiveness and poor prognosis (195). By contrast, high neutrophil counts in gastric tumors correlate with favorable prognosis (196). Since neutrophils constitute an already mature population that does not proliferate, possible changes in their metabolism have not been studied in depth. Regarding their metabolism, neutrophils are strongly committed to glycolysis and PPP, whereas their few mitochondria are used for maintenance of the redox balance. It has been shown that their high rates of glycolysis are necessary for the generation of ATP, in which HIF-1α is critically involved by regulating the expression of key glycolytic enzymes (197). Apart from its main role in energy metabolism, glycolysis has been shown to be essential for some neutrophil functions such as oxidative burst and chemotaxis (198). Indeed, Thompson et al. reported that murine HIF-2α deficient inflammatory neutrophils displayed no impairment of chemotaxis, phagocytosis, or respiratory burst but elevated sensitivity to apoptosis leading to reduced neutrophilic inflammation (199). It has also been shown that hypoxia can promote neutrophil recruitment by modifying the adherence properties of ECs to neutrophils (200). In addition, FAS has been reported to have some relevance in neutrophil biology. In this regard, Lodhi et al. showed that peroxisomal lipid synthesis drives inflammation by supporting neutrophil membrane phospholipid composition as well as viability (201). Another important feature of neutrophils is the formation of neutrophil extracellular traps (NETs). Brinkmann et al. described for the first time that activated neutrophils are able to release their chromatin (DNA and histones) loaded with granule enzymes forming an extracellular mesh-like structure that can both trap and kill extracellular organisms (202). Glucose uptake, glycolysis, and a shift toward PPP have been shown to be essential for NETs formation (203, 204). Beyond their bactericidal role, NETs has been described to sequester tumor cells and promote metastasis (205). Also, association of adhesion molecule and cytokines to NETs has been related to cancer-induced organ failure (206).

### Therapy Perspectives

Regarding tumor therapy, there are different approaches that can be used, including targeting of the cancer cells, or switching the nature of the immune cell to a more anti-tumoral state. During the past decade, a lot of attention has been given to try and selectively kill the tumor based on their metabolic alterations (207, 208). Indeed, increasing evidence supports the idea that dysregulated cellular metabolism is connected to drug resistance during cancer therapy. Therefore, combining cellular metabolism inhibitors with chemotherapeutic drugs constitutes a promising strategy to overcome this.

It has also become clear that there is much more the only Warburg effect when it comes to the metabolic rearrangements associated with malignant transformations. Indeed, there is also an increased flux through the PPP, higher rates of glutamine consumption and lipid biosynthesis, maintenance of redox homeostasis and limited levels of autophagy, at least in the first steps of oncogenesis (209–211).

#### Glycose Metabolism

Due to the high glycolic rate in developing tumors, regulation of this pathway in cancer cells has been considerately studied. Starting by targeting glucose intake, the GLUT1 inhibitor WZb117 has been shown to reduce ATP production and ER stress induction in cancer cells, with a synergistic anticancer effect in combination with cisplatin or paclitaxel (212). Also, under hypoxia conditions, Cao et al. reported that the GLUT1 inhibitor phloretin significantly enhances anticancer effects of the antibiotic daunorubicin, overcoming hypoxia-conferred drug resistance (213). In the same line, inhibition of glycolysis with 2-DG (glucose analog for hexokinase) in combination with radiation or chemotherapy treatments, enhance clinical efficacy of the latter (214). A great number of studies have focused on the importance that the highly expressed PKM2 enzyme in cancer cells has in conferring resistance to therapy (48, 215–217) (Figure 2). Inhibition of this last rate-limiting enzyme in the glycolytic pathway, increases apoptosis and inhibits proliferation during cisplatin (218), and docetaxel (219) treatment. In the last step of the glycolytic pathway, LDHA expression and activity has been reported to be higher in Taxol-resistant breast cancer cells. Inhibition of LDHA by oxamate (a pyruvate analog) in combination with paclitaxel treatment has shown synergistic effect on taxol-resistant cells by promoting apoptosis (220). Regulating the shift between glycolysis and TCA, PDK inhibits PDH conversion of pyruvate to acetyl-CoA. Inhibition of PDK3 (functional of pyruvate isoform even in high concentration) has shown to diminish hypoxia-induced resistance in cervical and colon cancer (221, 222).

Recent evidence also indicates that modulation of immunometabolism plays an important role in controlling immune responses against cancer progression. Indeed, several studies have focused on targeting metabolic pathways to enhance T cell function and persistence. One of the most promising is the use of PD-1 blocking antibodies to rescue T cell glycolysis and enhance Teff functions (223). By contrast, inhibition of mTOR (224, 225) or AMPK (226, 227) has been shown to lead to controversial results.

#### Lipid Metabolism

Since proliferation of cells requires the generation of novel phospholipid membranes, targeting *de novo* lipogenesis or steroidogenesis would also be a potential anticancer therapy approach (68). Several enzymes involved in these synthesis pathways, including FASN (228, 229), ACLY (230), ACCs (231), choline kinase (232, 233), monoglyceride lipase (75), and HMGCR (234) have been ascribed critical roles in oncogenesis or tumor progression *in vivo*, yet have not been tested in clinical settings. Indeed, FAS is significantly upregulated and correlates with poor prognosis in many types of cancer. Therefore, it is not surprising that several FAS inhibitors, such as cerulenin, C75, orlistat, C93, or GSL 837149a, have shown anti-tumor activity (Figure 3). In addition, the combination of FAS inhibition with docetaxel (235), trastuzumab (236), or adriamycin (237) treatment increases therapy sensitivity in breast cancer. Cancer cell metabolism is also highly dependent on glutaminolysis. It has been shown that glutamine in combination with leucine activates mTORC1 by enhancing glutaminolysis and α-ketoglutarate production (238). Targeting glutaminolysis by using the mTORC1 inhibitor rapamycin has been reported to enhance cisplatin treatment in gastric cancer (239). Pharmacological inhibition of FAO functions in MDSC, averts immune inhibitory pathways and decreases the production of inhibitory cytokines. Consequently, blocking FAO postponed tumor growth in a T-cell-dependent way, and increased the anti-tumor effect after adoptive T cell treatment (182). COX-2 inhibitors or reducing COX-2 expression in 3LL cells, obstructed their capacity to induce arginase I in MDSC (240).

#### Amino Acid Metabolism

Inhibitors of folate metabolism, thymidine and deoxynucleotide synthesis and elongation of nucleic acid are the so-called antimetabolites and serve as standard chemotherapeutic regimens against many human neoplasms (241). Unfortunately, these agents are linked to toxicity in bone marrow and intestinal epithelium, as these are highly proliferating tissues. Compound 968 (242) and bis-2-(5-phenylacetamido-1,2,4-thiadiazol-2-yl)ethyl sulfide, two specific GLS inhibitors (243), diminish glutamine catabolism and delay tumor growth in models of cancer. Targeting glutamate conversion to α-ketoglutarate by aminotransferases also diminishes tumor growth (244, 245). Replenishing the TCA cycle intermediates by providing substrates, such as glutamine, sustains mitochondrial metabolism in tumor cells (63) (Figure 4).

#### Mitochondria Respiration

Many types of tumor are highly dependent on OxPhos for their ATP (246–248). Therefore, these cells are probably sensitive to treatments that reduce mitochondrial ATP production. Moreover, inhibiting this mitochondrial ATP production would synergize together with approaches that diminish glycolysis, including inhibitors of the PI3K signaling pathway (249). It has been shown that phenformin (biguanide) inhibits mitochondrial complex I and in that way exerts its anti-tumor effects in experimental cancer models (250). Metformin has also antineoplastic activity (251). This appears to be independent of glycemia (252) and might reflect the ability to preferentially kill cancer stem cells, block mitochondrial respiration, intensify glutamine addiction, or limit inflammatory responses driving tumor growth (253–256). Indeed, its action is to specifically inhibit Mitochondrial complex 1, which in turn activates AMPK as a consequence of ATP decrease (257).

## Conclusion

Our understanding of metabolic changes in cancer development has improved significantly over the past years. However, the influence of the hypoxia pathway proteins on the metabolic pathways in tumor cells and the TME is still not entirely known. In a vast amount of physiological as well as pathological situations, hypoxia-induced rewiring permits survival during metabolic stress. Conversely, this drives cancer progression, causing enhanced lethality due to resistance to therapy and greater metastatic potential. Therefore, more research is necessary to better understand hypoxia-induced alterations in cellular metabolism and eventually target these pathways, thereby eliminating malignant cells.

## Author Contributions

All authors listed have made a substantial, direct, and intellectual contribution to the work and approved it for publication.

## Conflict of Interest Statement

The authors declare that the research was conducted in the absence of any commercial or financial relationships that could be construed as a potential conflict of interest.
